# Clinical, Paraclinical, and Evolutionary Profiles of Kidney Failure in Gold Miners Hospitalized in a Nephrological Service in a Sub-Saharan African Country

**DOI:** 10.1155/2020/4282969

**Published:** 2020-02-14

**Authors:** Gérard Coulibaly, Gaoussou Sanou, Moumouni Sanon, Aïda H. Y. Lengani, Juste Y. Bonzi, Aoua Semde

**Affiliations:** ^1^Centre Hospitalier Universitaire Yalgado OUEDRAOGO, Service de Néphrologie et Hémodialyse, 03 BP 7022, Ouagadougou, Burkina Faso; ^2^Centre Hospitalier Universitaire Sourô SANOU, Service de Néphrologie et Dialyse, Bobo-Dioulasso, Burkina Faso

## Abstract

*Introduction*. The purpose of this preliminary study is to describe the clinical, paraclinical, and evolutionary profiles of gold miner patients with kidney failure hospitalized in the nephrology and haemodialysis service in the Yalgado Ouédraogo University Hospital of Ouagadougou (CHU-YO). *Patients and Methods*. This was a longitudinal and descriptive study with a retrospective collection of data for the period from February 1, 2013, to March 31, 2018. Included were all gold miner patients who stayed and worked at an artisanal gold mining site for at least three months and who were diagnosed with acute or chronic kidney failure during hospitalization in the nephrology service. We collected sociodemographic, clinical, and paraclinical variables at admission and then three months later.

**Results:**

We included 50 patients; all were male and the average age was 29.4 ± 7.7 years. All patients were exposed to mercury and/or cyanide for an average of 4.5 ± 2.8 years. The average consultation/referral time for patients at the CHU-YO was 25.4 ± 14.9 days. The average of creatininemia was 2338.0 ± 791.4 *μ*mol/L. Kidney failure was acute in five cases (10%) and chronic in the remaining 45 cases or 90%. Extrarenal purification was indicated in 43 cases (86%). It was not performed in nine of the 43 cases due to lack of financial resources for patients (six cases) or death prior to the onset of haemodialysis (three cases). Thirty-two of the 50 patients in the study (64% of cases) died.

**Conclusion:**

Chronic kidney failure in gold miners appears to be common and late-managed. A prospective study of kidney disease and its causes at gold mining sites and surrounding areas will assess the extent of the problem in the country and better clarify the prevention of these diseases in our country.

## 1. Introduction

Artisanal mining or gold mining is a type of mining that has a negative impact on the environment and also on human health [1]. In addition to the often severe trauma, medical somatic diseases of mainly toxic causes have been reported in gold miners [1, 2].

Among medical diseases, kidney disease is not uncommon. WHO in March 2017 drew attention to the kidney involvement of these toxics, ranging from proteinuria alone to kidney failure [3]. In the particular case of Burkina Faso, we have seen an increasing frequency of admissions of gold miners in hospital for kidney failure (KF) in the country's only nephrology service until 2017. This KF could be directly or indirectly related to the professional activity of these patients.

Specific studies on the kidney health consequences of gold mining are rare in Africa. To our knowledge, in Burkina Faso, no studies have yet been carried out on this subject. And yet, considering the high risk of water and soil pollution from gold mining sites and surrounding areas with mercury and cyanide [4], the prevalence of chronic kidney disease could explode in the medium to long term in the country. We undertook this preliminary descriptive work on the clinical, paraclinical, and evolutionary profiles of gold miner patients with KF with the aim of guiding future lines of research on this problem already emerging as a public health problem in Burkina Faso.

## 2. Patients and Methods

This was a longitudinal and descriptive study with a retrospective collection of data over the period from 1 February 2013 to 31 March 2018, that is, 62 months. It concerned all the gold miner patients who were hospitalized for a KF in the department of nephrology and haemodialysis of the university hospital Yalgado Ouédraogo (CHU-YO) during the period indicated above. Included were all patients:

- Having stayed and worked on an artisanal gold mining site for at least three months
- Who in whom the diagnosis of acute renal failure (ARF) or chronic (CRF) has been made

We collected sociodemographic variables, personal pathological antecedents, and clinical and paraclinical variables at admission and three months later. The extreme values of certain clinical and paraclinical variables were considered for the purpose of the classification of the RF or the determination of an evolutionary profile.

The data were analyzed using the Epi Info 6.7 software. Quantitative variables were expressed as mean ± standard deviation or their median and qualitative variables as numbers and percentages.

In the absence of a local ethics committee, we requested and obtained the authorization of the CHU-YO's Executive Director to carry out the study. Anonymity and confidentiality in the collection and processing of data have been respected.

In an operational way, we defined certain notions:

(i) The gold miner was a person who had been involved in the artisanal mining of gold (gold panning) for at least three months
(ii) Chronic kidney disease (CKD) has been defined according to KDIGO criteria; CRF corresponded to stages 3 to 5 of the CKD [5]
(iii) In the absence of anatomopathological data, the criteria for the diagnosis of nephropathies presumed responsible for CRF were based on a bundle of clinical, biological, and radiological arguments
(iv) The ARF was selected according to the following criteria:
  - The abrupt or very recent character of the symptomatology, in the absence of personal pathological antecedents or paraclinical elements that may suggest a CRF
  - Normalization of serum creatinine in less than three months of evolution according to laboratory standards
(v) We distinguished three stages of ARF severity according to AKIN [6] creatininemia variation
(vi) Renal function was classified as ARF in the following manner: renal function recovered (decreased creatininemia by 75 to 100% with respect to the maximum value), improved (decreased creatininemia by 25% to 75%), stable (creatininemia variation of less than 25%), or degraded (increase of initial creatininemia by more than 25%)
(vii) Consultation time period was the time elapsed between the onset of symptomatology and the first contact with the nephrology department

## 3. Results

### 3.1. Frequency

We collected a total of 52 files of gold miner patients who were selected for the diagnosis of ARF (acute renal failure) or CRF (chronic renal failure). This represented 0.01% of the 4452 patients hospitalized in the nephrology and haemodialysis service of the CHU-YO during the study period. Two patients with nonusable clinical and paraclinical information were not included. Thus, our study involved 50 patients or about 10 cases per year.

### 3.2. Sociodemographic Characteristics

The patients were all male. The average age was 29.4 ± 7.7 years. The most represented age group was 20 to 30 years (Table 1).

### 3.3. Exposure to Chemicals

The chemicals identified were mercury and cyanide. All patients were exposed to at least one of these chemicals. Thirty-five patients (70%) used mercury alone. The use of cyanide alone was noted in seven patients (14% of patients). Eight patients (16%) used both mercury and cyanide. The number of years of exposure to mercury and/or cyanide was found in 44 patients (88.0%). The average duration of exposure to these products was 4.5 ± 2.8 years.

### 3.4. Clinical Data at Admission

The average patient consultation time was 25.4 ± 14.9 days. Fatigue was the most frequent functional sign on admission (41 cases or 82%) (Table 2).

Conjunctival paleness was found in 38 cases (76%). The mean blood pressure was 148.7 ± 47.4 mm Hg for the systolic and 87.5 ± 30.1 for the diastolic (Table 3).

Blood pressure was normal in 12 patients (24%). Low blood pressure was noted in three patients, or 6%. Thirty-five patients (70%) were hypertensive patients.

### 3.5. Biological Data at Admission

Creatininemia was high in all patients upon admission (Table 4).

Cytobacteriological examination of urine was performed in 29 patients (58%). It has successfully isolated a germ in seven cases (24.3%). The isolated germs were *Escherichia coli* (four cases), *Klebsiella pneumoniae* (two cases), and *Staphylococcus aureus* (one case).

The results of abdominal ultrasound were available in 45 patients (90%). No cases of dilatation of renal pelvis and calyces were noted.

### 3.6. Kidney Function

All 50 gold miner patients had impaired kidney function at admission to the service. Kidney failure was acute in five cases (10%) and chronic in the remaining 45 cases (90%). Various characteristics of these kidney failure patients are shown in Table 5.

### 3.7. Evolutionary Stage of Kidney Failure

In the case of ARF, the severity was stage 2 for two patients and stage 3 for the remaining three ARF patients. As for the CRF, it was moderate in two cases: 4.4% of the 45 patients in CRF; severe in four cases: 8.9%; and terminal in 39 cases: 86.7%.

### 3.8. Probable Injury Diagnosis of Kidney Failure

ARF was organic in the five patients who had it. They were divided into two cases of acute glomerulonephritis and three cases of acute tubular necrosis.

In the case of CRF, the probable injury diagnosis was as follows:

- A chronic glomerular nephropathy in 17 out of 45 cases (37.8%)
- A chronic tubulointerstitial nephropathy in 11 cases (24.5%)
- A chronic vascular nephropathy in one case (2.2%)
- A polycystic kidney disease in one case (2.2%)
- A chronic nephropathy undetermined in 15 cases (33.3%)

### 3.9. Evolutionary Aspects of Kidney Failure

The average length of stay in hospital for the 50 patients was 14.9 ± 8.3 days. It was 7.3 ± 3 days in patients with ARF and 20.2 ± 2 days for CRF cases. Renal function has evolved as follows in patients with ARF:

- A degradation in three cases
- An improvement in one case
- A stabilization in the fifth case

Extrarenal purification by haemodialysis was indicated in 43 cases (four cases of ARF and 39 cases of CRF) out of 50 (86%). The indication was as follows:

- Threatening hyperkalemia (two cases) and stage 3 AKIN (two cases) in patients with ARF.
- The end stage in 39 patients with CRF. Haemodialysis was urgently needed in 22 out of 39 cases (56.4%). The reason for this emergency was uremic encephalopathy (11 cases out of 22; 50%), severe presumed metabolic acidosis (seven cases, 31.8%), and acute pulmonary edema (four cases, 18.2%).

Haemodialysis was performed in 34 of the 43 patients (79.1%) to whom it was indicated. In the remaining nine cases, it could not be carried out due to the lack of financial resources of the patients (six cases) or their death before the onset of haemodialysis (three cases).

### 3.10. Death

Thirty-two of the 50 patients in the study, or 64% of cases, died. These were two cases of ARF and 30 cases of CRF. The average age of the 32 patients who died was 30.3 ± 8.6 years. In the case of ARF, the median age of death was 32.5 years, and in patients with CRF, the average age was 29.4 ± 6.2 years. The average creatininemia of deceased patients was 2568.0 ± 757.4 *μ*mo/L. The presumed causes of death were as follows:

- Severe pulmonary acute oedema in two cases, or 6.3% of the 32 deaths
- Heart failure in eight cases (25%)
- Severe sepsis in 10 cases (31.2%)
- Indeterminate in 12 patients (37.5%)

## 4. Discussion

We received 50 gold miners in hospital with a RF that was chronic and already severe at admission in 45 cases, in the only nephrology service in the country until 2017. This frequency, which we consider to be high, is most likely underestimated—CRF can remain symptomatically silent for a long time—if we refer to the national level because not all cases reach hospitals. This means that the prevalence of chronic kidney disease is most likely higher in gold mining areas than in the general population of Burkina Faso.

Mercury and cyanide are chemicals that are constantly found in artisanal gold mining [7, 8]. Mercury use was more frequently (70%) found in our patients. These products are recognized as nephrotoxic [7, 9]. The lesional mechanism of these toxic substances on the kidney is insufficiently described in the literature. Mercury may be responsible for pathological proteinuria, haematuria, and kidney failure [10, 11]. Cyanide, with the exception of mercurial cyanide salts that have the same renal toxicity of inorganic mercury, hydrogen cyanide, and its salts have no direct renal toxicity [12].

Gold miners are constantly exposed to breathed silica. The latter's responsibility for the occurrence of chronic kidney disease has been established without the mechanism of kidney damage being elucidated. These are essentially chronic tubulointerstitial or glomerular nephropathy. In the latter case, the few studies available on the subject did not eliminate confounding factors [13–15].

Kidney function, with an average creatininemia of 2338.0 ± 791.4 *μ*mol/L, was severely impaired in the subjects of our study. We have not found studies in the rest of the world that indicate such severe impairment of kidney function in gold miners. This severity of KF in our patients clearly reflects a late delay in their management and also explains the high death rate. This late delay can have several origins: low geographical accessibility to health facilities, self-medication very common at gold mining sites, and deplorable living and hygiene conditions [16]. It may also be patient ignorance or delay in diagnosis or referral by an underqualified health worker. Indeed, in rural areas in Burkina Faso, care is provided by paramedics in the health facilities of the first rung of the health pyramid.

The most common suspected chronic kidney disease was glomerular (37.8%) and tubulointerstitial (24.4%). Occupational kidney disease of toxic origin is more often tubulointerstitial than glomerular [13, 17]. However, mercury is more likely to be responsible for chronic glomerular kidney diseases [18]. The higher frequency of mercury use (70%) by the subjects of our study most likely contributes to the prevalence of chronic glomerular nephropathy. We also cannot rule out other etiological factors, including infectious factors, in a population with a high risk of infection, including HIV infection [19].

### 4.1. Limitations and Constraints of Study

Our study is a hospital-based one and its results cannot be extrapolated to the general population of gold miners. Moreover, the absence of histological and toxicological data does not make it possible to be precise about basic kidney disease and their possible toxic origin. Nevertheless, this study has the merit of reporting tropical data on a subject still understudied in Africa while gold mining activity is increasing in volume in African countries in general, in Burkina Faso in particular.

## 5. Conclusion

Chronic kidney failure seems common in the gold mining population and most likely in the general population surrounding gold mining sites due to the high risk of water and soil contamination. Most gold miner patients arrive late in the nephrology service, in the terminal stage of the CRF. Burkina Faso's various health actors should initiate or intensify the early detection and management of CKD at gold mining sites, as well as awareness-raising and prevention of communicable diseases and risks associated with the safe use of conventionally incriminated toxics. A prospective study of kidney disease and its causes at gold mining sites and surrounding areas will assess the extent of the problem in the country and better identify prevention areas.

## Figures and Tables

**Table 1:** Frequency of sociodemographic characteristics of 50 gold miner patients with kidney failure.

Sociodemographic variable	*n* (%)
Age	
<20 years	3 (6)
20–30 years	28 (56)
30–40 years	14 (28)
≥40 years	5 (10)
Origin	
Rural	43 (86)
Urban	7 (14)
Professional activity	
Nothing but gold mining	29 (58)
Other activity^*∗*^	21 (42)

^*∗*^Agriculture (16 cases), small business (3 cases), and carpentry (2 cases).

**Table 2:** Consultation time period and functional signs found in 50 gold miner patients with kidney failure.

Variable	Result
Consultation time period	
Average ± standard deviation (days)	25.4 ± 14.9
<7 days, *n* (%)	4 (8)
(7–14) days, *n* (%)	8 (16)
(14–21) days, *n* (%)	13 (26)
≥21 days, *n* (%)	25 (50)
Functional signs at admission	
Fatigue, *n* (%)	41 (82)
Lack of appetite, *n* (%)	39 (78)
Vomiting, *n* (%)	35 (70)
Oliguria, *n* (%)	25 (50)
Abdominal pain, *n* (%)	24 (48)
Effort dyspnoea, *n* (%)	22 (44)
Headaches, *n* (%)	21 (42)
Anuria, *n* (%)	14 (28)
Lumbar pain, *n* (%)	12 (24)
Dizziness, *n* (%)	10 (20)
Cough, *n* (%)	11 (22)
Fever, *n* (%)	11 (22)
Chest pain, *n* (%)	10 (20)
Others, *n* (%)	4 (8)

**Table 3:** General and physical signs found in 50 gold miner patients with kidney failure.

Variable	Result
General signs	
Conjunctival paleness, *n* (%)	38 (76)
Lower legs oedema, *n* (%)	33 (66)
Facial puffiness, *n* (%)	23 (46)
Alteration of general condition, *n* (%)	6 (12)
Skin fold of dehydration, *n* (%)	5 (10)
Average systolic blood pressure (mm Hg)	148 .7 ± 47.4
Average diastolic blood pressure (mm Hg)	87.5 ± 30.1
Physical signs/syndromes	
Tachycardia, *n* (%)	16 (32)
Jugular veins turgescence, *n* (%)	8 (16)
Ascites, *n* (%)	3 (6)
Pulmonary acute oedema, *n* (%)	2 (4)
Distended bladder, *n* (%)	1 (2)

**Table 4:** Average value of various biochemical and haematological analyses performed at admission in 50 gold miner patients with kidney failure.

Biological analysis	*n* (%)	Average ± SD	Range
Plasma urea (mmol/L)	50 (100)	40.9 ± 14.8	10.3–83.0

Creatininemia (*μ*mol/L)	50 (100)	2338.0 ± 791.4	594–4651

Uricemia (*μ*mol/L)	28 (56)	705.7 ± 211.5	435–1281

Natremia (mmol/L)	50 (100)	131.5 ± 9.1	109–146

Kaliemia (mmol/L)	50 (100)	5.0 ± 1.4	2.6–7.5

Bicarbonatemia (mmol/L)	36 (72)	18.4 ± 5.4	7–29

Calcemia (mmol/L)	50 (100)	1.7 ± 0.4	0.9–2.3

Phosphataemia (mmol/L)	48 (96)	3.0 ± 0.9	0.7–5.3

Protidemia (g/L)	39 (78)	64.9 ± 9.1	51–89

Proteinuria (g/24 hours)	50 (100)	2.0 ± 0.9	0.5–4.7

Hemoglobinemia (g/dL)	50 (100)	8.6 ± 2.3	4.2–14.5

Rate of leukocytes (el/mm^3^)	50 (100)	10672.2 ± 3456	4600–18523

Rate of platelets (el/mm^3^)	50 (100)	162283 ± 3824	88000–410000

SD: standard deviation; el: elements; *n*: number of patients for whom test results were available.

**Table 5:** Sociodemographic, clinical, and paraclinical data by type of kidney failure in 50 gold miner patients.

	Acute kidney	Chronic kidney
	failure, *n* = 5	failure, *n* = 45
Age		
Average in years, *a* ± SD	34.4 ± 16.9	28.9 ± 6.1
Use of chemicals		
Mercury alone, *n* (%)	3	32 (71.1)
Cyanide alone, *n* (%)	1	6 (13.3)
Mercury and cyanide, *n* (%)	1	7 (15.6)
Exposure time in years	Median = 3.5	*a* ±SD = 4.4 ± 2.1
Consultation time period		
Median in days	10.5	—
Average in days, *a* ± SD	—	26.9 ± 14.9
Biological data		
Creatininemia in *μ*mol/L	1498.6 ± 529.7	2814.1 ± 854.7
Plasma urea in mmol/L	11.2 ± 6.8	32.3 ± 19.4
Natremia in mmol/L	136.5 ± 9.1	126.5 ± 9.3
Kalemia in mmol/L	4.0 ± 1.4	4.8 ± 1.7
Bicarbonatemia in mmol/L^*∗*^	19.4 ± 5.4	16.2 ± 9.2
Calcemia in mmol/L	1.8 ± 0.4	1.6 ± 0.4
Hemoglobinemia in g/dL	12.6 ± 2.3	8.6 ± 4.3

*a* ± SD: average ± standard deviation; *n*: the number of patients for whom the results of the examination were available. ^*∗*^*n* = 31.

## Data Availability

The data used to support the findings of this study are available from the corresponding author upon request.
